# Alterations in Cardiovascular Parameters in 5xFAD Murine Model

**DOI:** 10.1002/cbf.70138

**Published:** 2025-11-10

**Authors:** Andrea G. Marshall, Dominique Stephens, Kit Neikirk, Sepiso K. Masenga, Bryanna Shao, Amber Crabtree, Zer Vue, Heather K. Beasley, Edgar Garza‐Lopez, Estevão Scudese, Benjamin I. Rodriguez, Han Le, Steven Damo, George E. Taffet, Okwute M. Ochayi, Ronald McMillan, Jeremiah M. Afolabi, Vernat Exil, Ashton Oliver, Vineeta Sharma, Pamela Martin, Amadou Gaye, Chanel Harris, Briar Tomeau, LaCara Bell, Markis′ Hamilton, Taneisha Gillyard, Railyn Webster, Marcus Jackson, Prem Prakash, Shanilah Frierson, Chandravanu Dash, Margaret Mungai, Annet Kirabo, Jennifer A. Gaddy, Nelson Wandira, Antentor Hinton, Anilkumar K. Reddy

**Affiliations:** ^1^ Department of Molecular Physiology and Biophysics Vanderbilt University Nashville Tennessee USA; ^2^ Department of Life and Physical Sciences Fisk University Nashville Tennessee USA; ^3^ Department of Cardiovascular Science and Metabolic Diseases Livingstone Center for Prevention and Translational Science Livingstone Southern Province Zambia; ^4^ Department of Internal Medicine University of Iowa Iowa City Iowa USA; ^5^ Laboratory of Biosciences of Human Motricity (LABIMH) of the Federal University of State of Rio de Janeiro (UNIRIO) Rio de Janeiro Brazil; ^6^ Sport Sciences and Exercise Laboratory (LaCEE) Catholic University of Petrópolis (UCP) Petrópolis Rio de Janeiro Brazil; ^7^ Department of Medicine Houston Methodist Hospital Houston Texas USA; ^8^ Department of Physiology Faculty of Basic Medical Sciences, College of Medicine and Health Science, Baze University Abuja Federal Capital Territory Nigeria; ^9^ Department of Medicine Vanderbilt University Medical Center Nashville Tennessee USA; ^10^ Department of Pediatrics, Division of Cardiology St. Louis University School of Medicine St. Louis Missouri USA; ^11^ Vanderbilt Center for Immunobiology, Vanderbilt University Nashville Tennessee USA; ^12^ Vanderbilt Institute for Infection Immunology and Inflammation, Vanderbilt University Nashville Tennessee USA; ^13^ Department of Biomedical Sciences Meharry Medical College Nashville Tennessee USA; ^14^ The Center for AIDS Health Disparities Research (P.P., R.C., G.M., M.B., C.D.), Meharry, Medical College Nashville Tennessee USA; ^15^ Department of Microbiology Immunology, and Physiology (P.P., R.C., G.M., M.B., C.D.), Meharry Medical College Nashville Tennessee USA; ^16^ Department of Biochemistry Cancer Biology, Pharmacology and Neuroscience (P.P., R.C. G.M., M.B., C.D.), Meharry Medical College Nashville Tennessee USA; ^17^ Vanderbilt Institute for Global Health Vanderbilt University Nashville Tennessee USA; ^18^ Department of Pathology Microbiology, and Immunology, Vanderbilt University Medical Center Nashville Tennessee USA; ^19^ Department of Medicine Division of Infectious Diseases, Vanderbilt University Medical Center Nashville Tennessee USA; ^20^ Department of Medicine Health, and Society, Vanderbilt University Nashville Tennessee USA; ^21^ Department of Veterans Affairs Tennessee Valley Health Systems Nashville Tennessee USA; ^22^ Department of Public Health School of Health Sciences Busoga University Iganga Iganga Eastern Region Uganda; ^23^ Department of Medicine Baylor College of Medicine Houston Texas USA; ^24^ Indus Instruments Webster Texas USA

**Keywords:** 5xFAD, alzheimer's disease, cardiovascular disease, pulse wave velocity

## Abstract

Alzheimer's Disease (AD) is a global health issue, affecting over 6 million people in the United States, with that number expected to increase as the population ages. As a neurodegenerative disorder that affects memory and cognitive functions, it is well established that AD is associated with cardiovascular risk factors beyond only cerebral decline. In this study, we measured hemodynamic parameters related to cardiovascular and cerebrovascular function in 5xFAD mice with AD and their littermates. Specifically, we measured cardiovascular pulse wave velocity parameters, a marker of arterial stiffness and cardiovascular risk, and cerebrovascular pulse wave velocity, a novel technique to measure cerebral arterial stiffness. Our results showed that while 5xFAD mice exhibited significant differences in ejection time, pulse pressure, and Tei index, many other cardiovascular and cerebral parameters were not different. Despite reports that amyloid plaque deposition begins at an early age of 1.5 months in 5xFAD mice, our results did not indicate significant cardiovascular changes. Studies to elucidate cardiovascular and cerebrovascular parametric changes should be done at later ages where the underlying changes are more profound.

## Summary

- Alzheimer's Disease (AD) and cardiovascular dysfunction are critically linked, but the timing and nature of these interactions remain poorly understood.
- This study introduces Cerebral Pulse Wave Velocity as a novel, noninvasive method to assess cerebral arterial stiffness, enhancing the rigor of combined cardiovascular and cerebrovascular research.
- By demonstrating that significant hemodynamic changes are absent in young 5xFAD mice despite early amyloid pathology, our work establishes a crucial timeline and suggests that cardiovascular decline may be a consequence of later‐stage disease.
- This provides a foundation for future studies to target specific ages and mechanisms linking vascular health to AD progression.

## 1 Introduction

Alzheimer's disease (AD) is a progressive neurodegenerative disorder that affects millions of people worldwide and causes deficits in quality of life, marked by a decline of memory and cognitive functions [1]. AD is a critical disease to investigate, with an estimated yearly burden amounting to $305 billion which is expected only to grow with the aging population [1]. A hallmark feature of AD is the accumulation of aggregated amyloid beta (Aβ) protein in the brain, which ultimately leads to synaptic dysfunction, neuronal loss, and cognitive impairment [2]. In addition to genetic factors, lifestyle and environmental factors such as stress and diet have been implicated in the development and progression of AD [3]. Beyond dementia, AD also impacts cardiovascular function, as the Aβ plaques also accumulate in the heart [4]. A key risk factor of AD is hypertension (HTN), and it has been hypothesized that neuroinflammatory effects link these pathologies [5].

AD is well understood to cause neuropsychologic decline but is also understood to affect cardiovascular hemodynamics. AD importantly is shown to potentially be associated with HTN through the formation of Aβ plaques in HTN, which could limit cerebral blood flow [6]. In human populations, even after adjusting for risk factors such as age, a reduction in HTN is correlated with increased retention of cognitive ability, suggesting blood pressure treatment as a potential mechanism for managing AD [4]. This connection between blood pressure and AD is particularly evident in relation to systolic pressure [7]. However, a previous review on the topic suggests that the issue is far more complex, as many past studies have not used standardized HTN measurements. There may also be age‐ and ethnicity‐dependent influences on the relationship between HTN and AD development [8]. Together, these findings highlight the need for a deeper understanding of hemodynamics in AD.

Our goal was to investigate cardiovascular‐related parameters in 5xFAD mice, which carry five AD‐linked mutations. The 5xFAD mouse model expresses human amyloid precursor protein (APP) and presenilin 1 (PS1) mutations, resulting in the rapid accumulation of Aβ and the development of AD‐like pathology [9]. This model also shares significant similarities with human disease models, including the Aβ‐butyrylcholinesterase association and key hallmarks such as progressive loss of cognitive function accompanied by reduced synaptic markers [10]. Due to apoptotic neuron loss, 5xFAD mice display memory deficits by 4 months of age [11]. The development of disease at a young age allows discrimination between age‐associated arterial stiffening and that associated with AD. This mouse model has been widely used to study the mechanisms underlying AD and to test potential therapeutic interventions.

The full extent of the mechanisms which link AD and cardiovascular disease remains poorly understood. In this study we sought to determine the basic understanding of how AD alters cardiovascular and cerebral hemodynamics.

## 2 Methods

### 2.1 Animals

All animal protocols were approved by the Institutional Animal Care and Use Committee of Baylor College of Medicine in accordance with the National Institutes of Health Guide for the Care and Use of Laboratory Animals. We used 5 male and 5 female littermate controls (C57BL/6J‐WT) and male (*n* = 10) and female (*n* = 10) 5xFAD mice at 2–3 months of age. Diets of both groups of mice consisted of standard commercial chow (2920X Harlan Teklad, Indianapolis, IN, USA) with free access to food and water. Anesthesia was induced in mice at 2.5% isoflurane initially and then were transferred to a heated (37°C ± 1°C) electrocardiography board (MouseMonitor S, Indus Instruments, Webster, TX) where anesthesia was maintained with 1.5% isoflurane via nose cone. The paws of the mice were taped to the four electrodes to measure electrocardiogram (ECG).

### 2.2 Doppler Flow Velocity Measurements

We used a 20 MHz Doppler probe to measure doppler aortic outflow velocity, mitral inflow velocity, and carotid flow velocity signals. The signals were measured sequentially with the same probe used at different arterial and cardiac sites, acquired and stored using Doppler Flow Velocity System (DFVS; Indus Instruments, Webster, TX) along with blood pressure and ECG signals. We measured peak and mean aortic velocities, stroke distance (Sd), and aortic ejection time (ET) from aortic outflow velocity, and peak‐E (early) velocities, isovolumic contraction time (IVCT), isovolumic relaxation time (IVRT) from mitral inflow signals, and myocardial performance index (MPI, also known as Tei index = (IVCT + IVRT)/ET). From the carotid flow velocity signal, we calculated peak, minimum, and mean velocities, and pulsatility and resistivity indices.

### 2.3 Blood Pressure Measurements

Blood pressure measurements were made as previously described [12, 13, 14], Briefly, a 1‐French (0.33 mm diameter) blood pressure catheter (SPR‐1,000: Millar Instruments Inc., Houston, TX) was introduced via the isolated right carotid artery and advanced into the ascending aorta to measure aortic pressure. About 2–3 s segments of blood pressure signals were acquired (simultaneously with either aortic flow velocity or carotid flow velocity and ECG signals) with the DFVS system. Systolic (SBP), diastolic (DBP), mean (MBP), pulse pressures (PP = SBP‐DBP), end‐systolic pressure (ESP) and rate‐pressure product (RPP) were calculated from the recorded aortic blood pressure signals.

### 2.4 Determination of Aortic and Cerebral Impedance

The method to determine aortic impedance was described elsewhere [12, 14, 15, 16]. Aortic impedance was determined using the aortic pressure‐velocity relationship. The foot of the aortic pressure waveform was aligned with the foot of the aortic velocity waveform to avoid potential errors in the phase relation between pressure and velocity signals. The signals are converted to the frequency domain using fast Fourier transform and impedance ( | Z|= |P | / | V | ) parameters (peripheral vascular resistance [aZ_0_], characteristic impedance [aZ_C_], and impedance at first harmonic [aZ_1_]) are calculated. Aortic pulse wave velocity was calculated as aZ_C_/ρ (ρ‐density of blood). The foot of the blood pressure waveform was aligned with the foot of carotid flow velocity waveform to determine cerebral impedance in the same way as aortic impedance. The cerebral impedance parameters (cerebral vascular resistance [cZ_0_], cerebral characteristic impedance [cZ_C_], and cerebral impedance at first harmonic [cZ_1_]) were calculated. Cerebral pulse wave velocity was calculated as cZ_C_/*ρ* (*ρ*‐density of blood).

### 2.5 Calculation of Parameters to Determine Ventriculo‐Vascular Coupling

Elastance was determined as previously discussed [12, 17]. Arterial elastance (Ea) was calculated as ESP/SV (stroke volume, SV = Sd * aortic cross‐sectional area) and stroke work (SW) was calculated as ESP*SV [17].

The definition of hemodynamic parameters is detailed below (Table 1).

**Table 1:** Definitions of hemodynamic parameters.

Measurement	Definition
Aortic cross‐sectional area	The area of the aortic lumen, used with stroke distance to calculate stroke volume.
Aortic ejection time	Represents time period of blood ejection into the aorta during systole, used to assess left ventricular systolic function.
Aortic elastance	Stiffness of the aorta and its resistance to blood ejection during systole.
Aortic pulse wave velocity	Velocity of pressure or flow waves traveling through the aorta; index of aortic stiffness.
Body weight	The overall mass of mice, as measured in grams.
Cerebral pulse wave velocity	Velocity of pressure or flow waves traveling from the carotid artery to the cerebral arteries, indicator of arterial stiffness in the brain.
Cerebral cZ_1_	Impedance at first harmonic in indicating the influence of wave reflectionsfrom cerebral circulation.
Cerebral cZ_C_	The characteristic impedance of the cerebral circulation, reflects the stiffness of the cerebral arteries.
Cerebral cZ_0_	Measure of cerebrovascular resistance which is an indicator of the opposition to blood flow in cerebral vasculature.
Deceleration time	Time for the early (E) mitral velocity to decelerate to zero flow during diastole.
End systolic pressure	Residual pressure in the heart at the end of systolic period used to assess cardiac output.
Heart rate (HR)	Beats per minute (bpm) general characteristic typically known to decrease with age and rudimentary indicator of cardiovascular health.
Impedance at first harmonic (aZ_1_)	Blood flow resistance at the frequency of the first harmonic of the pulse wave, which is an indicator of aortic wall stiffness.
Impedance‐based cerebral (cPWV_Z_)	Cerebral PWV calculated using cZ_C_, a measurement of cerebral arterial stiffness.
Impedance‐based pulse wave velocity (aPWV_Z_)	Aortic PWV calculated using Z_C_, a measurement of aortic stiffness.
Isovolumic contraction time	Length of time between mitral valve closing and aortic valve opening.
Isovolumic relaxation time	Length of time between aortic valve closing and mitral valve opening.
Mean carotid velocity	Average velocity of blood flow in the carotid artery.
Mean aortic outflow velocity	Average velocity of blood flow in the aorta.
Minimum carotid flow velocity	The minimum velocity of blood flow through the carotid artery.
Myocardial performance index (Tei index)	A measure of the overall function of the heart, which takes into account systolic and diastolic function, as calculated by (IVCT + IVRT)/ET.
Peak aortic outflow velocity	Maximum velocity of blood flow during systole in the aorta.
Peak carotid velocity	Maximum velocity of blood flow in the carotid artery during systole.
Peak mitral‐early flow velocity	Maximum velocity of blood flow in early diastole.
Pulsatility index (PI)	Also known as the Gosling index, calculated by (Vmax ‐ Vmin)/Mean velocity, and indicator of vascular resistance.
Resistivity index (RI)	Also known as the Pourcelot index, resistance to blood flow calculated by (Vmax ‐ Vmin)/Vmax which serves as an indicator of vascular resistance.
Stroke distance	The distance the blood travels during a cardiac cycle, which is an indicator of the heart's stroke volume.
Stroke work	Also known as cardiac work, stroke volume multiplied by mean aortic pressure, which assesses the amount of work done by the heart during a cardiac cycle.
Total peripheral resistance (aZ_0_)	Resistance to blood flow in the systemic circulation, as measured by combined effects of factors including blood vessel diameter and blood viscosity. Indicator of overall cardiovascular health.
Systolic blood pressure	Peak blood pressure during the systolic phase in a cardiac cycle, key measurement for factors including hypertension.
Diastolic blood pressure	Minimum blood pressure during the diastolic phase in a cardiac cycle, key measurement for factors including hypertension.
Mean blood pressure	Average arterial pressure during the cardiac cycle, including systole and diastole phases, representative of systemic vascular resistance.
Pulse pressure	Difference between systolic and diastolic blood pressure, representative of risk factors for arterial stiffness and cardiovascular disease.
Rate pressure product	Systolic pressure multiplied by heart rate, an indicator of cardiac stress.

### 2.6 Statistical Analyses

All the data are presented as mean ± standard error of the mean (SEM). Statistical analyses were performed using an unpaired *T*‐test to compare conditions in each sex through Prism (GraphPad Software; La Jolla, USA).

## 3 Result

Our analyses revealed similar trends in female 5xFAD vs. female control mice in comparison to male 5xFAD vs. male controls. So, we combined data from both genders and summarized as shown in Table 2.

**Table 2:** Summary of (male and female) combined data of 5xFAD mice and their littermate controls.

Parameter (units)	WT Mice	5xFAD Mice	*p*‐value
	Average ± SEM	Average ± SEM	
*General parameters*	*n* = 10	*n* = 20	
BW (g)	35.2 ± 2.2	26.9 ± 0.9	0.0040
Aortic cross‐sectional area (cm^2^)	0.0118 ± 0.0005	0.0097 ± 0.0003	0.0036
Heart rate (bpm)	389 ± 12	535 ± 11	0.0000
*Aortic flow velocity*			
Peak aortic velocity (cm/s)	115.3 ± 4.5	113.1 ± 4.6	0.730
Mean aortic velocity (cm/s)	28.3 ± 1.6	32.4 ± 1.3	0.056
Aortic stroke distance (cm)	3.5 ± 0.13	3.7 ± 0.16	0.322
Aortic ejection time (ms)	56.0 ± 1.4	44.6 ± 1.3	0.0000
*Carotid flow velocity*			
Peak carotid velocity (cm/s)	57.2 ± 3.8	55.8 ± 2.2	0.764
Minimum carotid velocity (cm/s)	8.48 ± 0.75	9.45 ± 0.75	0.367
Mean carotid velocity (cm/s)	19.3 ± 1.0	20.2 ± 1.0	0.494
Pulsatility index	2.54 ± 0.20	2.34 ± 0.11	0.404
Resistivity index	0.80 ± 0.02	0.83 ± 0.01	0.748
*Aortic blood pressure*			
Systolic pressure (mmHg)	102.0 ± 2.2	102.4 ± 3.3	0.916
Diastolic pressure (mmHg)	80.1 ± 2.0	84.9 ± 3.0	0.193
Mean pressure (mmHg)	90.5 ± 2.0	93.6 ± 3.1	0.418
Pulse pressure (mmHg)	21.9 ± 0.5	17.5 ± 0.6	0.0000
Rate x pressure (bpm·mmHg)	39673 ± 1511	54745 ± 2030	0.0000
*Aortic impedance*			
aZ_0_ (dyne·s/cm^3^)	8860 ± 425	8137 ± 390	0.223
aZ_1_ (dyne·s/cm^3^)	428 ± 32	371 ± 20	0.147
aZ_C_ (dyne·s/cm^3^)	305 ± 39	288 ± 16	0.695
aPWV_Z_ (cm/s)	309 ± 36	271 ± 15	0.350
*Cerebral impedance*			
cZ_0_ (dyne·s/cm^3^)	5902 ± 325	6837 ± 543	0.151
cZ_1_ (dyne·s/cm^3^)	776 ± 60	782 ± 63	0.948
cZ_C_ (dyne·s/cm^3^)	358 ± 37	325 ± 22	0.456
cPWV_Z_ (cm/s)	338 ± 35	307 ± 21	0.455
*Ventricular‐vascular coupling*			
End systolic pressure (mmHg)	91.8 ± 2.0	92.2 ± 3.0	0.916
Aortic elastance (mmHg/μL)	2.32 ± 0.16	2.73 ± 0.18	0.103
Stroke work (mmHg·μL)	3814 ± 324	3346 ± 249	0.266
*Mitral flow velocity*			
E‐peak velocity (cm/s)	67.9 ± 2.0	77.6 ± 2.6	0.0071
Isovolumic contraction time (ms)	18.4 ± 1.3	10.3 ± 0.4	0.0001
Isovolumic relaxation time (ms)	22.7 ± 1.0	14.9 ± 0.5	0.0000
Myocardial performance or Tei index	0.73 ± 0.03	0.57 ± 0.02	0.0012

**Figure 1:**
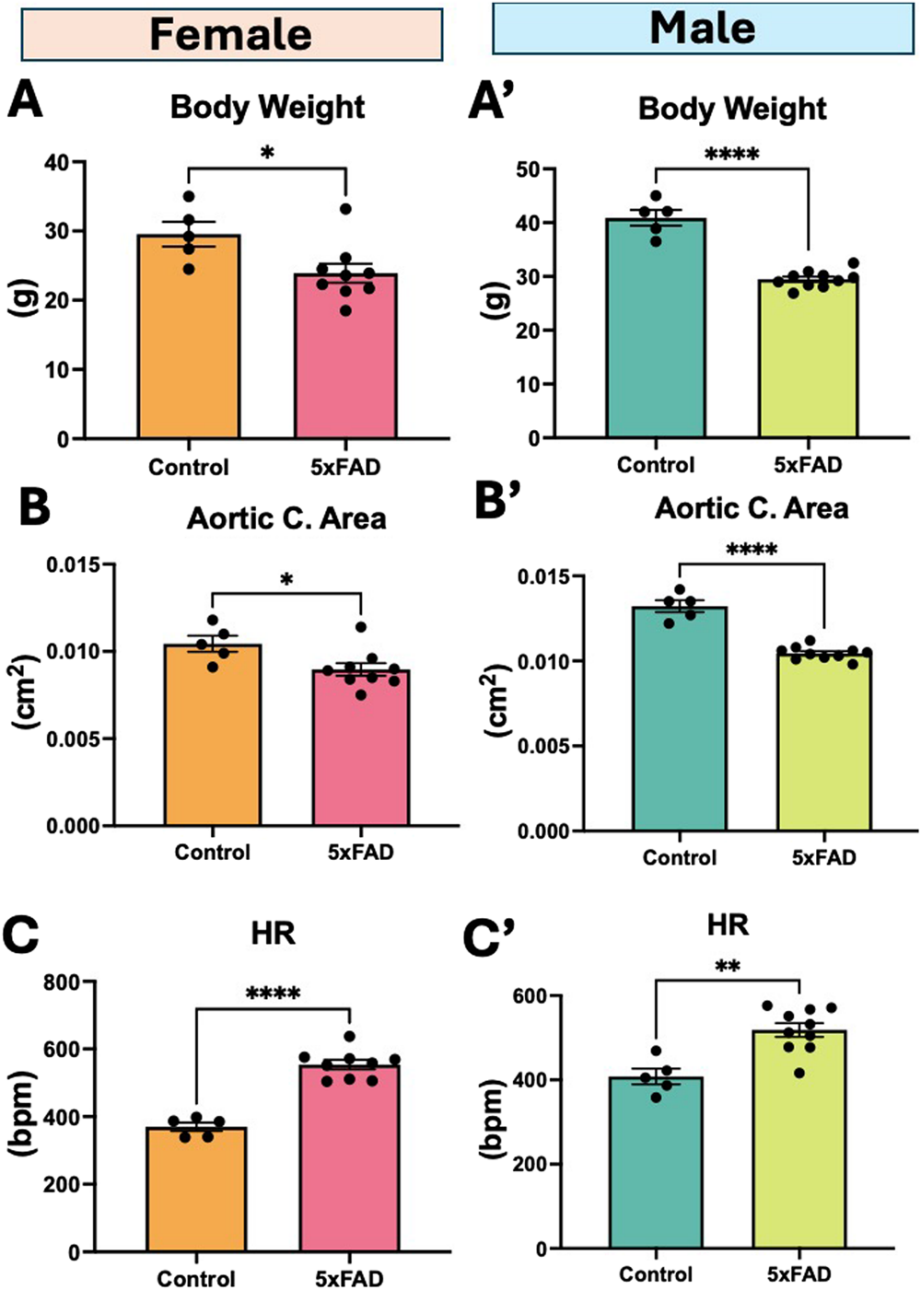
General parameters in wild‐type and 5xFAD mice. (A,A′) Body weight, (B,B′) aortic cross‐sectional area, and (C,C′) heart rate (HR) in male and female mice. Data are mean ± SEM (*n* = 5–10/group). *p < 0.05, **p < 0.01, ****p < 0.0001, ns = not significant by unpaired *t*‐test.

The general parameters of body weight and aortic cross‐sectional area were significantly lower in 5xFAD mice compared to control (Figure 1A,B′), while heart rates (HR) were significantly higher in 5xFAD mice (Figure 1C,C′).

**Figure 2:**
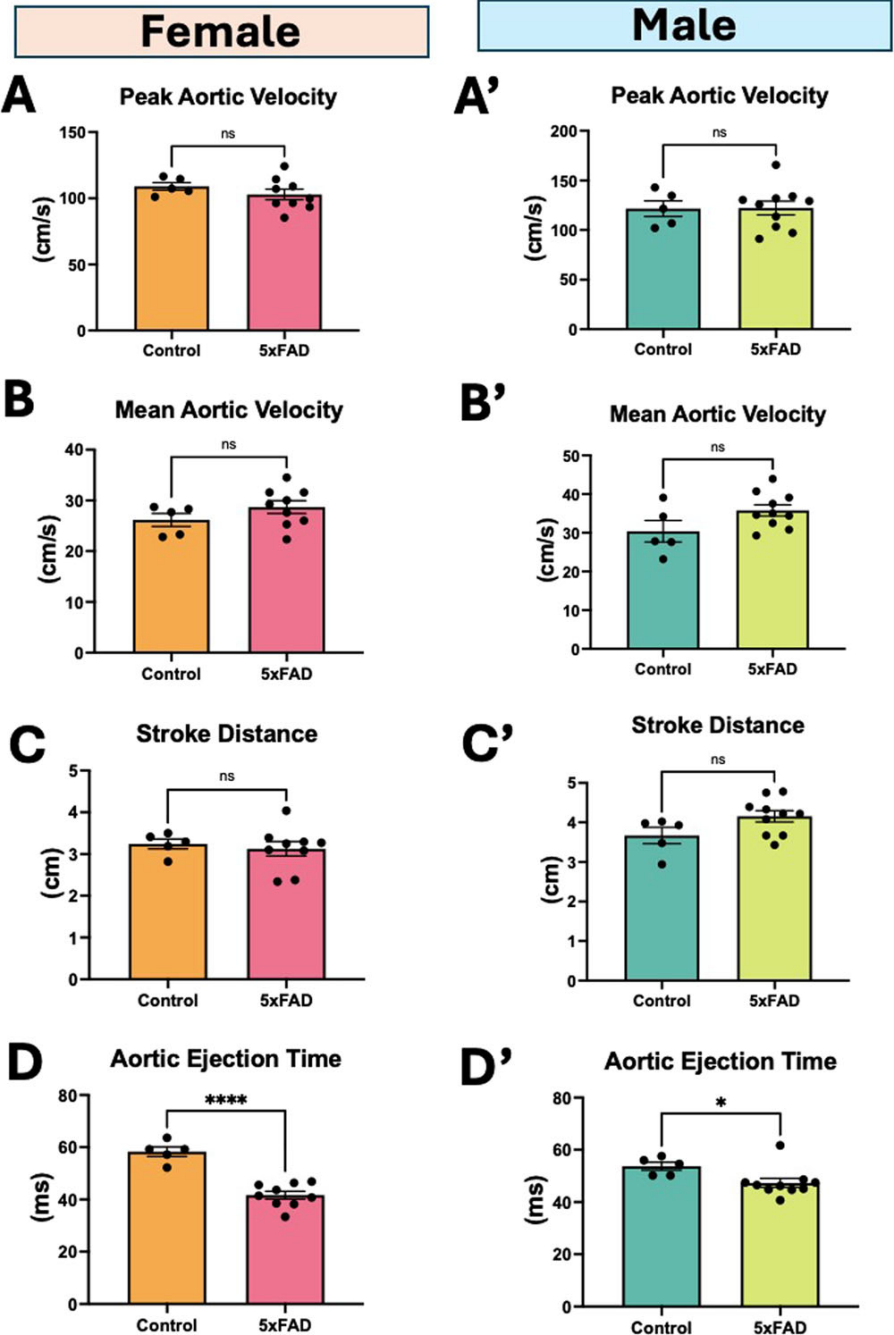
Aortic flow velocity parameters. (A,A′) Peak aortic outflow velocity, (B,B′) mean aortic flow velocity, (C,C′) aortic stroke distance, and (D,D′) aortic ejection time in male and female mice. Data are mean ± SEM (*n* = 5–10/group). *p < 0.05, **p < 0.01, ****p < 0.0001, ns = not significant by unpaired *t*‐test.

Using aortic flow velocity to measure cardiac systolic function, we found that 5xFAD mice demonstrated lower aortic ejection times (Figure 2D,D′). However, there was no significant differences in peak aortic velocity, mean aortic velocity, and stroke distance (Figure 2A–C′).

For the carotid flow velocity data, there no significant differences observed in either the peak, mean, and minimum carotid artery velocities or in the pulsatility index (PI) and resistivity index (RI) (Table 2) between the groups.

**Figure 3:**
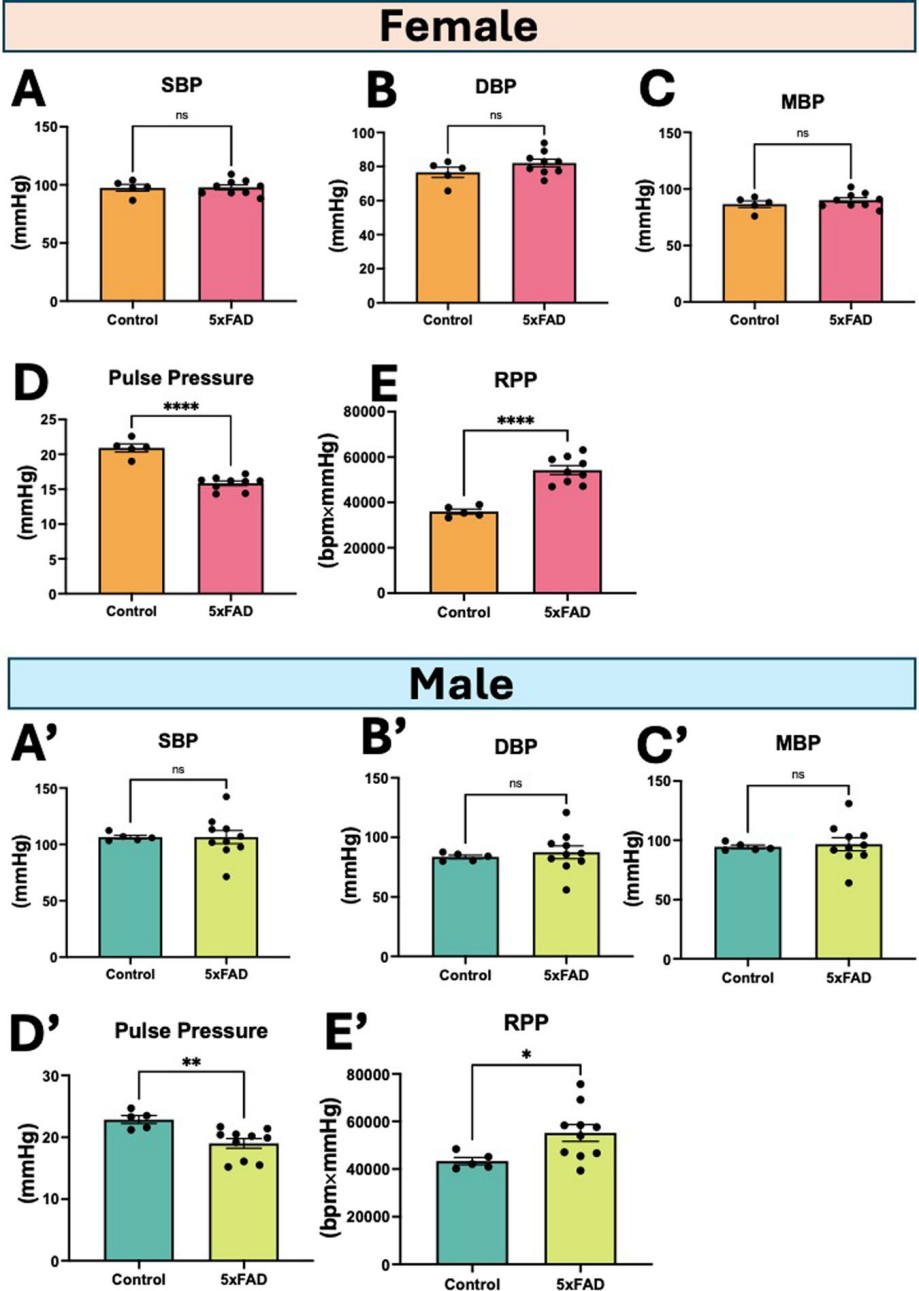
Aortic blood pressure parameters. (A,A′) Systolic blood pressure (SBP), (B,B′) diastolic blood pressure (DBP), (C,C′) mean blood pressure (MBP), (D,D′) pulse pressure, and (E,E′) rate pressure product (RPP = HR × SBP) in male and female mice. Data are mean ± SEM (*n* = 5–10/group). *p < 0.05, **p < 0.01, ****p < 0.0001, ns = not significant by unpaired *t*‐test.

In the aortic blood pressure data, we observed no significant differences in systolic, diastolic, and mean blood pressures (Figure 3A–C′) between the groups, but we found that pulse pressure (Figure 3D,D′) was significantly lower and rate‐pressure product RPP (Figure 3E,E′) was significantly higher in 5xFAD mice.

No significant differences were observed in the parameters of aortic impedance (aZ_0_, aZ_1_, aZ_C_, or aPWV_Z_) or cerebral impedances (cZ_0_, cZ_1_, cZ_C_, or cPWV_Z_) (Table 2).

The ventricular‐vascular coupling parameters of end systolic pressure, arterial elastance, stroke work, and stroke distance exhibited no differences (Table 2) between the groups.

**Figure 4:**
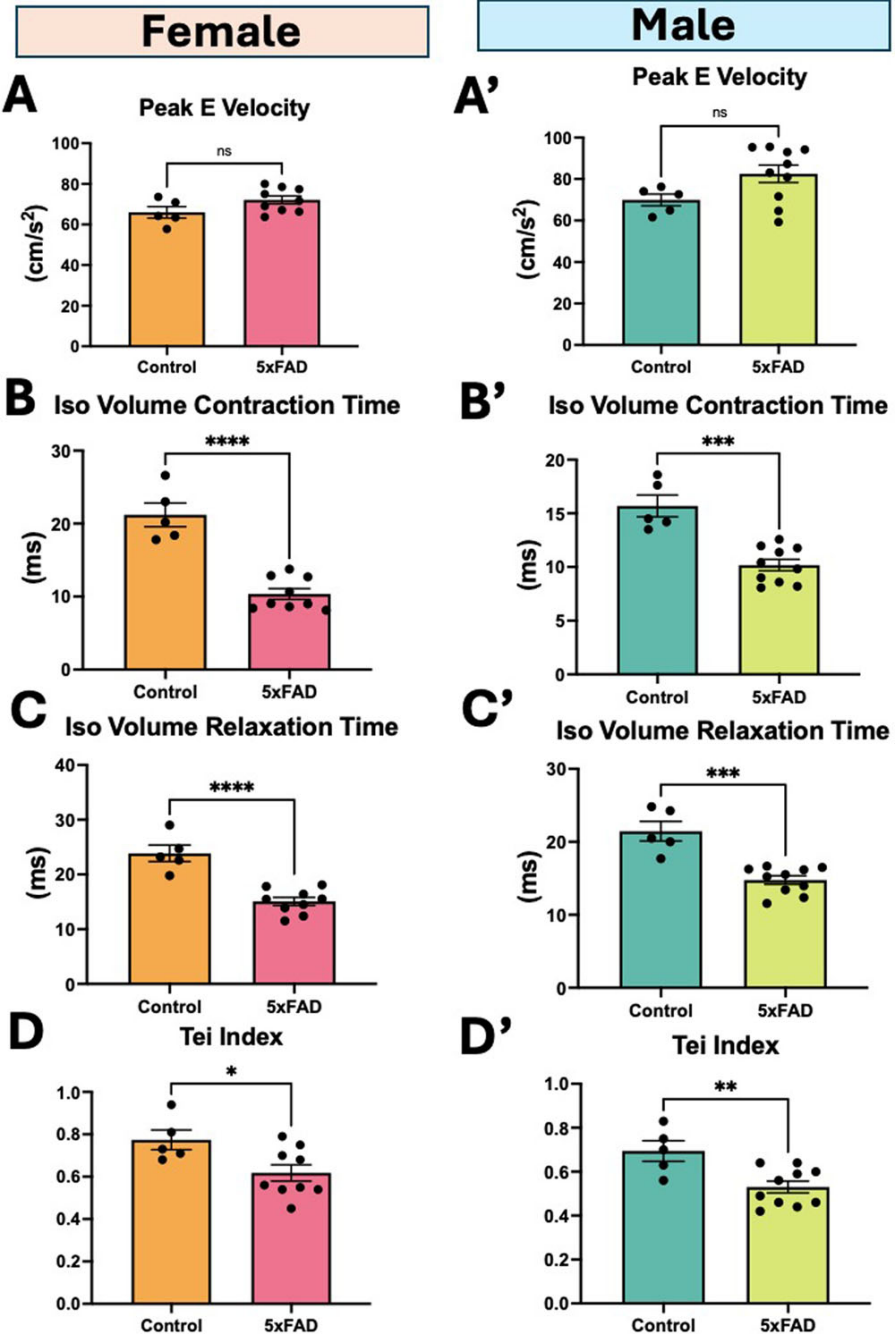
Mitral flow velocity parameters. (A,A′) Mitral peak‐early flow velocity (E wave), (B,B′) isovolumic contraction time (IVCT), (C,C′) isovolumic relaxation time (IVRT), and (D,D′) myocardial performance index (Tei index). Data are mean ± SEM (*n* = 5–10/group). *p < 0.05, **p < 0.01, ****p < 0.0001, ns = not significant by unpaired *t*‐test.

Mitral flow velocity measurements showed that the E‐peak velocity (Figure 4A,A′) was significantly higher in 5xFAD mice, and isovolumic contraction time, isovolumic relaxation time, and myocardial performance (Tei) index (Figure 4B–D′) were all significantly lower.

## 4 Discussion

The goal of this study was to characterize early vascular changes that may indicate the progression of AD. Here, we characterized cerebral, cardiac, and aortic hemodynamics in 5xFAD mice at an early age with hope of catching the initiation time point in AD progression. From the general parameters, we found that 5xFAD mice were of smaller size than controls and hence had lower aortic cross‐sectional area. This perhaps resulted in higher heart rates in these mice owing to the mammalian scaling factor of smaller sized animals having higher heart rates [18]. In an echocardiography study involving 5xFAD mice, heart rates have not been reported [19]. However, based on the one set of m‐mode images provided, we determined that the heart rate in the 2‐month‐old mice 5xFAD was about the same in WT (assuming same time scale). This is unlike the heart rates seen in our 5xFAD mice. In general, isoflurane mildly depresses the heart rate, which perhaps was not the case in 5xFAD mice in our study.

### 4.1 Aortic and Carotid Blood Flow Velocity

The cardiac systolic function as determined by aortic flow velocity parameters did not show any significant differences except aortic ejection times (Figure 2D), which may have resulted from higher heart rates in 5xFAD mice. It is known that shorter left ventricle ejection time concomitantly occurs with impaired ejection fraction and stroke volume [20] which is in agreement with the findings by Murphy et al. [19], who reported that 2‐month old 5xFAD mice had lower ejection fraction. This could be due to pathological changes beginning at early age and resulting in amyloid‐protein deposition at 2 months of age in 5xFAD mice [21, 22]. The carotid flow velocity had no significant changes in any of the parameters (Table 2), indicating that perhaps the cerebral function is either not compromised or may have triggered myogenic autoregulatory responses [23] to maintain cerebral circulation.

Past studies have shown that Aβ deposition typically increases alongside blood pressure in human models [8], so future studies may aid to understand how in the 5xFAD model, which shows no change in overall blood pressure at an early time point, changes in Aβ deposition lead to blood pressure and cardiovascular changes with age.

### 4.2 Aortic Blood Pressure

While no significant changes were observed in in the systolic, diastolic, and mean blood pressures in 5xFAD mice, we found that pulse pressure was significantly lower, which may indicate reduced cardiac output (lower ejection fraction reported by Murphy et al. [19]), resulting in increased heart rate in the 5xFAD mice. We found that pulse pressure in both groups was less than 25% of systolic blood pressure. It is reported that pulse below 25% of the systolic pressure typically occurs with cardiac dysfunction, aortic stenosis, cardiac tamponade, or blood loss [24], but this may occur in aged animals and not in 2–3 month mice. Lower pulse pressures may be associated with poor cerebral blood flow leading to Alzheimer's disease or dementia [25] which could explain the lower pulse pressures observed in the 5xFAD mice of our study. In humans, mild to moderate AD is associated with reduced pulse pressure and impaired cerebral blood flow, suggesting these may be early maladaptations that contribute to later pathology [26]. We also found that rate × pressure product (RPP) was significantly higher in the 5xFAD mice. An indicator of myocardial oxygen utilization, RPP is reflective of overall cardiac workload [27], which may explain the higher workload due to higher pumping rate of the heart to make up for lower cardiac output.

### 4.3 Aortic and Cerebral Impedance

When evaluating the aortic peripheral vascular resistance (aZ_0_,), strength of wave reflections from the periphery (aZ_1_), characteristic impedance (aZ_C_), and aPWV_Z_ we did not find significant differences in 5xFAD mice compared to their controls. Akin to our previous findings [13], it would be expected that pulse pressure and ejection time would concomitantly change with aZ_C_ and aPWV_Z_ as the aortic stiffness index should correlate between these measures. Yet, we did not observe this in these groups of young mice.

Similarly, we did not observe significant differences between the groups in cerebral impedance parameters. Past studies implicated increased arterial stiffness to negative cerebral outcomes in a linear fashion with age [28].

### 4.4 Ventricular‐Vascular Coupling

With respect to ventricular vascular coupling, we did not observe any differences in the end systolic pressure. While aortic elastance was higher and stroke work was lower in the 5xFAD mice, they did not reach the level of significance. Higher aortic elastance may indicate increased vascular load that affects ventricular‐vascular coupling [29]. In the study reported by Murphy et al. [19], the normal functioning cardiomyocytes of 5xFAD mice had a significant reduction in sarcomere shortening leading to contractile function impairment perhaps leading to reduced stroke work.

### 4.5 Mitral Flow Velocity

Significantly higher peak‐E velocity reflects rapid filling during early diastolic phase which could be indicate normal diastolic function or restrictive filling diastolic dysfunction as determined by peak‐E/peak‐A (atrial) velocity. In our study, however, we could not reliably measure peak‐A velocity due to fusing of A‐wave with E‐wave caused by high heart rates. IVCT, the time from mitral valve closing to aortic valve opening, and IVRT, the time from aortic valve closing to mitral valve opening, are both associated with LV ejection time (ET). Myocardial performance index (or Tei Index) that is calculated from IVRT, IVCT & ET provides an overall systolic and diastolic performance independent on ventricular geometry [20]. The 5xFAD mice had a significantly lower IVRT, IVCT & ET that resulted in a significantly lower Tei Index, indicating better cardiac function compared to control mice. The lower Tei index is confounding given that 5xFAD mice had lower ejection fraction and reduced sarcomere shortening [19]. Therefore, Tei index calculation perhaps using the tissue Doppler indices may be needed to confirm the above finding.

## 5 Conclusion

In conclusion, our study showed no significant changes in most of the hemodynamic parameters examined. However, significant differences were observed in the body weights, aortic cross‐sectional area, ejection time, pulse pressure, rate × pressure product, mitral peak‐E velocity, IVCT, IVRT, and ET. These findings agreed with those reported in 5xFAD and their littermate controls. The aortic and cerebral impedances findings suggest that the relationship between PWV and AD pathology in animal models may have to be looked at later ages of these mice. Future studies are needed to elucidate the underlying mechanisms and their implications for AD development and progression.

### 5.1 Limitations

There are several limitations to this study that must be considered when interpreting the results. While 5xFAD mice serve as a strong model for human AD, they lack neurofibrillary tangles which humans have in AD [9]. Another limitation of our study is that we did not measure Aβ deposition levels, which may be used to be correlate with PWV [30], or other molecular markers of AD pathology. Therefore, it is possible that our findings do not fully capture the complex relationship between hemodynamic parameters and AD pathology. Finally, our study only examined one‐time point (at 12 weeks of age), and it is possible that with aging the 5xFAD mice may have different effects on hemodynamic parameters and AD pathology.

## Author Contributions

Equal contribution: A.G.M., D.S., K.N., and S.K.M. contributed equally to data collection, experimentation, data analysis, manuscript review, and editing. Conceptualization: S.K.M., A.G.M., D.S., K.N., A.K.R., A.H. Methodology: A.G.M., S.K.M., D.S., K.N., B.I.R., H.L., A.H., A.K.R. Validation: A.H., A.K.R. Formal analysis: A.G.M., D.S., K.N., S.K.M., B.S., A.K.R., A.H. Investigation: A.G.M., D.S., K.N., S.K.M., B.S., B.I.R., A.H., A.K.R. Resources: A.K., S.K.M., A.H., A.K.R. Data curation: A.G.M., D.S., K.N., S.K.M. Writing – Original Draft: A.G.M., D.S., K.N., S.K.M., A.H., A.K.R. Writing – Review and Editing: All authors. Supervision: S.K.M., A.K.R., A.H. Project administration: S.K.M. Funding acquisition: S.K.M., A.K.R., A.H.

## Disclosure

This study was done when Dr. Reddy was faculty at Baylor College of Medicine. He is now a Principal Investigator at Indus Instruments, Webster, TX. He is also a Visiting Scientist at the Houston Methodist Research Institute; The authors declare no conflicts of interest.

## Data Availability

The datasets generated and/or analyzed during the current study are available from the corresponding author on reasonable request. All data supporting the findings of this study are included within the article and its supplementary materials, unless otherwise stated.
